# Serum Biochemistry of Greater Rhea (*Rhea americana*) in Captivity in the Northeast of Brazil

**DOI:** 10.3390/ani13132103

**Published:** 2023-06-25

**Authors:** Antonio Humberto Hamad Minervino, Carolina A. S. C. Araújo, Herbert S. Soares, Eloine M. B. Picanço, Yasmine R. Batista Silva, Clara Satsuki Mori, Solange Maria Gennari, Raimundo Alves Barrêto Júnior, Enrico Lippi Ortolani

**Affiliations:** 1 Laboratory of Animal Health (LARSANA), Federal University of Western Pará (UFOPA), Santarém 68040-470, PA, Brazil; 2 Faculdade de Medicina Veterinária e Zootecnia, Universidade de São Paulo, São Paulo 05508-270, SP, Brazil; 3 Programa de Pós-Graduação em Biociências, Federal University of Western Pará (UFOPA), Santarém 68040-470, PA, Brazil; 4 Department of Animal Science, Federal Rural University of the Semiarid Region (UFERSA), Mossoró 59625-900, RN, Brazil

**Keywords:** biochemical profile, Brazil, captivity, *Rhea americana*

## Abstract

**Simple Summary:**

The greater rhea (Rhea americana) is the largest endemic bird species in the central and northeastern regions of Brazil. It has economic importance due to its meat, leather, and feathers being appreciated, but its hunting is only allowed in registered commercial breeding grounds because of its threat of extinction. In Brazil, these animals are found mainly in captivity, thus facing different management and nutrition than free-living animals, a fact that can affect health problems identified only through laboratory tests. This study carried out an analysis of the serum biochemistry of greater rhea raised in captivity in the northeast of the country and identified that the values of calcium, cholesterol, and uric acid were higher in females than in males, allowing the result to be attributed to diet or stress at the time of collection. Our study is the first report on the biochemical profile of greater rheas in captivity in Brazil, and the data showed broad-spectrum results which contribute to the diagnosis of diseases through the analysis and interpretation of the biochemical profile of these animals in the practice of the veterinary clinic.

**Abstract:**

We investigated the biochemical profile of greater rheas (*Rhea americana*) in captivity and correlated these values according to the birds’ sex. A total of 69 serum samples were collected from a breeding site in Mossoró, northeastern Brazil, and analyzed to quantify serum biochemical parameters (total protein, albumin, cholesterol, calcium, phosphorus, uric acid, urea, creatinine, ALP, AST, and CK). The birds had levels of urea, creatinine, total cholesterol, aspartate aminotransferase, calcium, and phosphorus similar to the values reported for ratite and ostrich species. By sex, females showed higher values (*p* < 0.05) of calcium (3.5 mmol/L), total cholesterol (7.5 mmol/L), and uric acid (435.3 μmol/L) than males, which had 3.1 mmol/L, 3.8 mmol/L, and 390.7 μmol/L, respectively. This can be attributed to the difference in diet, the productive phase of females, or stress at the time of sampling. The data present a wide spectrum of biochemical results regarding the health of greater rheas, contributing to the veterinary clinical practice of this species.

## 1. Introduction

The *Rhea americana* is a large flightless bird of the Rheidae family, popularly known as rheas or greater rhea [1,2]. It is endemic to South America and inhabits the open areas of eastern and central Brazil, Paraguay, Uruguay, and northern and central Argentina, preferring higher grasslands for nesting [3,4]. They live in mixed groups of up to 50 individuals with males and females of all ages. *R. americana* have many similarities with the ostrich (*Struthio camelus*); however, they are smaller birds [5,6]. The greater rhea is the largest bird in South America, reaching an average weight of 20 to 27 kg in adulthood and 127 to 140 cm in length from beak to tail. Life expectancy in the wild is about 10 to 15 years. It is an omnivorous bird but mainly herbivorous, eating a wide variety of plant matter (leaves, seeds, fruits, and flowers), invertebrates, and small vertebrates [7].

Rheas are threatened with extinction due to unregulated trade, destruction, fragmentation, or loss of their habitat to other animals, especially mammals [4,8]. The greater rhea is listed as Near Threatened in the IUCN Red List of Threatened Species [9]. Over the years, rheas populations have been harmed by human exploitation because of their great economic potential, as they produce meat, leather, and feathers of excellent quality, contributing to the development of many rural and urban areas [10]. However, during the 1990s, captive breeding of this species expanded mainly in the United States, Canada, and some European countries, having gained importance as an agricultural and subsistence activity, as well as for recreational use in countries such as Argentina, Uruguay, and Brazil [11].

In Brazil, the hunting of rheas is controlled, and the bird is under government protection; the marketing of products that originate from these animals, such as meat, is only allowed from registered commercial breeding facilities [6,12]. Rheas raised in captivity face different management and diets in relation to free-living animals, which may lead to clinical problems [4,13]. Information concerning the physiological values of plasma clinical chemistry variables of wildlife is important for breeders to access the health status of animals, and it may provide clues to improve captivity diets [4,14]. 

Considering the limited information available regarding the clinical chemistry profile of greater rhea, we aimed to determine the biochemical variable range in a population of greater rheas bred in captivity in the state of Mossoró, Rio Grande do Norte, Brazil.

## 2. Materials and Methods

The present study was carried out in a breeding site (5°12′48″ S, 37°18′36″ W) in the municipality of Mossoró, RN, northeast region of Brazil. Serum samples were collected from 69 adult *R. americana*, 24 males and 45 females, with a mean age of 4.3 years (ranging from 3 to 8 years). Each bird was clinically examined as described [15] and manually restrained, without the use of any type of anesthetic drug. The birds were sampled in the beginning of the morning before feeding and underwent 8–10 h of fasting prior to sampling. The animals were not in active laying during sampling.

Blood samples were collected from the basilic vein and placed in tubes without anticoagulant for biochemical determinations. After blood harvesting, the animals were identified with an open stainless steel ring around the leg and carefully returned to the breeding site. Serum was stored at −20 °C for a maximum period of 6 months until the biochemical analyses. 

Serum samples were analyzed for total protein, albumin, total cholesterol, calcium, phosphorus, uric acid, urea, and creatinine and for the activity of the enzymes alkaline phosphatase (ALP), aspartate aminotransferase (AST), and creatine kinase (CK) in an automated biochemical analyzer (model Daytona, Randox Laboratories Ltd., Antrim, UK) using commercial kits (Randox^®^ Laboratories Ltd., Antrim, UK) according to the manufacturer´s recommendations. All biochemical parameters performed followed the guidelines for evaluating bird health [15] and standard laboratory practices [16]. 

Statistical analysis was performed using Student’s t-test (two-sample t-test) to compare the results of each of the biochemical parameters in relation to the sex. *p* < 0.05 was considered significant.

## 3. Results

Table 1 presents the results of biochemical analysis including the range and reference range for each variable when possible. For the total protein and albumin concentrations, rheas showed 4.9 (±0.57) and 2.6 (±0.32) g/dL, respectively; cholesterol presented values of 6.2 ± 3.7 mmol/L. The mean serum concentrations of calcium and phosphorus were, respectively, 3.3 ± 0.52 mmol/L and 1.3 ± 0.21 mmol/L. The mean serum uric acid concentration in the rheas was 419.2 ± 91.9 mmol/L. As for urea, the value found was 0.98 ± 0.58 μmol/L, and for creatinine, the value found in this study was 2.9 ± 5.64 μmol/L.

In birds, liver function can be assessed by evaluating liver enzyme activity that may reflect hepatocellular damage, with the most used enzymes being AST and ALP [2]. The mean serum activities of the rheas’ enzymes were 38.2 ± 24.0 UI for AST, 91.5 ± 276.4 UI for ALP, and 207.4 ± 224.3 for CK. 

Table 2 shows the statistical analyses of each biochemical variable in relation to the rheas’ sex. No differences were found between male and female birds regarding PT, albumin, phosphorus, urea, creatinine, AST, LDL, and CK. Males had lower (*p* < 0.05) serum concentrations of uric acid, cholesterol, and calcium in relation to females.

Table 3 contains the biochemical parameters found in our study with greater rhea (*R. americana*) in comparison with biochemical parameters found in studies with greater rhea and ostriches (*Struthio camelus*). 

## 4. Discussions

Blood parameters are influenced by many factors, including age, condition, sex, nutrition, and disease. Clinical conditions in birds are sometimes difficult to identify, as physical examination is limited in providing a detailed diagnosis. Evaluation of the serum biochemical profile provides useful information about their physical condition, making it a useful tool in differentiating apparently healthy birds from abnormal or diseased ones. Blood test results can attest to the condition of a particular individual and can be used to recognize many diseases [22,23].

As for serum proteins, total protein (TP) values were similar to those elucidated by [20] in birds and by [24] in their research with adult emus (*Dromaius novaehollandiae*) (4.83 ± 0.7). The authors of [25] pointed out that the age factor is directly related to the values of this parameter, because young birds have higher TP values, given that this nutrient is related to the growth and development of muscles and feathers. Serum albumin was higher than the reference range and that reported by [25] in their research, which was 3.58 ± 0.05 in ostriches (*Struthio camelus*). In [26], with broilers, serum albumin levels ranged from 0.5 to 1.4 ± 0.1 g/dL. Albumin may be slightly increased in the blood of birds during egg formation, which could alter the albumin/globulin ratio, a protein found in the blood. This change, however, is not indicative of injuries and is considered normal [27].

These values may be altered in females because of the composition of the diet, or because they are ingesting more protein and excreting less bile acids [18,28]. Birds need a diet with a higher protein content in the initial phase of life in order to guarantee the development of various tissues and organs because, during this period, there is a high demand for nutrients, mainly proteins [29]. In the case of males, the demand is higher, which justifies the lower levels in relation to females [30].

As for cholesterol (6.2 ± 3.7 mmol/L), the variable presents values above the normality standards for the species [18], where 1.94 ± 0.13 mmol/L is considered a normal value. Cholesterol is part of the synthesis of steroid hormones and is associated with the nutritional management of animals and low protein consumption in the diet, as the reduced excretion of bile acids causes elevations in cholesterol concentrations [13], although studies indicate that lipemia is common in rheas [31]. Stress, endogenous corticosterone, and endogenous steroid hormones can cause elevations in cholesterol levels in Darwin’s rhea [18]. A survey on the biochemical profile of broiler birds carried out a comparison of age groups and sex of these species and reported lower values of uric acid and cholesterol in males [32].

Serum concentrations of low-density lipoproteins (LDL) and very-low-density lipoproteins (VLDL), associated with cholesterol transport, occur in much lower proportions in males than in females, compared to high-density lipoproteins [33]. The liver of birds is the largest producer of HDL, and within the Golgi apparatus, there are spherical and discoid forms of said lipoprotein, but in circulation, unlike mammals, only one form of HDL is known in birds, regardless of dietary status, line of cut, or sex. The functions of HDL are to accept apolipoproteins and phospholipids from degraded lipoproteins, accept cholesterol from tissues and lipoproteins, and deliver cholesterol esters to these to maintain the lipid/protein balance. These characteristics depend on the composition of the diet, which plays an important role in controlling blood cholesterol concentrations in birds [34,35].

The values of calcium and phosphorus are in agreement with research that evaluated ostriches aged 2 to 3 years [17]. Normal calcium concentrations in birds can reach values much higher than those tolerated in mammalian species, reaching concentrations of 30 g/L [36]. Higher values of calcium in females of the same age group compared to males could be associated with reproductive activity [37]. It has been suggested that the calcium level can be explained by increased secretion of steroid hormones [38].

Usually, the urea and creatinine values are measured for the evaluation of the renal function of domestic mammals, providing evidence for the diagnosis and/or prognosis of nephropathies [39]. Higher blood urea values may be the result of ingesting large amounts of animal protein. As they are primarily herbivorous, these birds tend to have lower urea values when compared to other species [13]. Unlike what happens in mammals, the variable of choice for monitoring kidney function in birds is uric acid [28,40]. Uric acid is the main product of protein metabolism, and its levels can be used as an indicator of dehydration and kidney disease. An increase in the concentration of uric acid in the blood of birds reaching values above 15 mg/dl can lead to uric gout, formation of urate deposits in the kidneys, urolithiasis, and renal damage with chronic failure of the organ, which can cause the death of the animal and also indicates protein overload in the diet. Although birds kept in captivity commonly present metabolic disorders, including uric gout syndrome, in conservation veterinary medicine, there is a need for non-invasive investigative methods, with practical application, for the diagnosis of these diseases. However, care must be taken if blood collection is performed using the fingernail, as contamination with excrements may occur, generating increased values [13,41,42].

The creatinine levels found (5.64 µmol/L) were well below the reference values found [19], which in turn have already been reported as being below the reference values of 17.7–29.0 µmol/L for the species [43]. The low levels of creatinine were attributed to the advanced age of the birds and to the smaller muscular volume in relation to the young birds [19]. GGT concentrations were higher than those found by [19], which corroborates the finding by [44] indicating that this enzyme can vary from 0 to 12.1 IU/L in greater rheas and ostriches. The GGT enzyme is altered in biochemical tests when liver injuries occur in these animals [45].

According to [19,46], analysis of the activities of enzymes in the blood is important in the diagnosis of pathologies, parasitism, and subclinical metabolic conditions; feeding practices; and welfare of rheas, and several of these enzymes activities are included in the study of the blood metabolic profile. 

In the previous study [18] with Darwin’s rhea (*Rhea pennata*), changes in AST activity were observed, and the authors suggested that it may be associated with excess glucocorticoids, which are directly linked to the biological response to stress and may suppress reproduction, growth, and immunity if activated recurrently [47]. Studying emus (*Dromaius novaehollandiae*) in Canada [19], it was observed that AST activity values in healthy animals were 179 IU/L. Plasma ALP activity is a non-sensitive specific test for hepatocellular disease in birds and has no advantages compared to AST as a test for hepatocellular disease [36]. Research on the effects of dietary fiber on the development and growth of geese in China [48] indicated that as the liver of birds functions more healthily, the activity of the ALP enzyme decreases.

The activity of the CK enzyme in birds is located primarily in muscle cells and is therefore an excellent indicator of muscle damage, and its elevation is related to muscle injury such as muscle wasting, rough handling, or trauma [28]. The increase in CK enzyme activity in Darwin’s rhea (*Rhea pennata*) may be associated with stress at the time of collection, which increases the concentrations of these metabolites [18].

During the development of this study, the lack of articles related to biochemical variables in greater rhea was identified, which makes this research relevant for the diagnosis of diseases through the analysis and interpretation of the biochemical profile of this species. Our results show biochemical results from one single sampling point of apparently healthy animals. A repeated sampling study on the same animals from different sites considering diet composition and clinical and behavioral information would be required to establish with more clarity the relationship between the biochemical results and the animals’ health status. 

## 5. Conclusions

To the best of our knowledge, this is the first report of the biochemical profile of greater rhea (*R. americana*) in captivity in Brazil. Analyses of serum biochemistry in greater rheas kept in captivity showed higher values of calcium, cholesterol, and uric acid in females than in males. The information presented in this article, although obtained from a relatively small number of animals, contributes to the veterinary clinical literature on greater rheas and provides valuable baseline serum biochemical parameters for comparison for this species.

## Figures and Tables

**Table 1:** Serum biochemistry parameters found in greater rheas (*R. americana*) maintained in captivity in northeast region of Brazil.

Parameters	Mean	SD	Range	Reference Interval
Total protein (g/dL)	4.9	0.57	3.99–6.46	3.4 ± 5.6 ^5^
Albumin (g/dL)	2.56	0.32	1.7–3.3	1.0 ± 2.5 ^5^
Cholesterol (mmol/L)	6.2	3.7	0.6–16.8	1.94 ±0.1 ^2^
Calcium (mmol/L)	3.3	0.52	2.25–5.85	3.85 ± 0.21 ^2^
Phosphorus (mmol/L)	1.3	0.21	0.74–1.71	1.9 ± 0.08 ^2^
Uric acid (µmol/L)	419.2	91.9	225.3–667.5	317. 67 ± 20.82 ^2^
Urea (mmol/L)	0.98	0.58	0.02–3.9	0.6 ± 0.30 ^1^
				0.8 ^4^
Creatinine (µmol/L)	29.6	5.64	15.9–45.97	12.0 ^4^
ALP (UI/L)	91.5	276.4	10.8–2248.0	171.5 ± 45.9 ^3^
AST (UI/L)	38.2	24	12.2–193.3	43.7 ± 7.3 ^2^
CK (UI/L)	207.4	224.3	29.2–1464.5	22.3 ± 3.1 ^2^
				933.0 ± 269.0 ^3^

SD: Standard deviation. ^1^ Verstappen et al. [17], ^2^ Reissig et al. [18], ^3^ Palomeque et al. [13], ^4^ Menon et al. [19], ^5^ Ritchie et al. [20].

**Table 2:** Serum biochemistry concentration and statistical comparison between sex in greater rheas (*R. americana*) maintained in captivity in the northeast region of Brazil.

Parameters	Male		Female		p *
	n	Mean	n	Mean	
Total protein (g/dL)	24	4.88	44	5.03	0.354
Albumin (g/dL)	24	26.8	45	25.0	0.023
Cholesterol (mmol/L)	24	3.8	45	7.5	<0.001
Calcium (mmol/L)	24	3.1	44	3.5	<0.001
Phosphorus (mmol/L)	24	1.31	44	1.30	0.888
Uric acid (μmol/L)	24	390.7	45	435.3	0.042
Urea (mmol/L)	24	1.0	42	0.89	0.353
Creatinine (μmol/L)	24	30.5	45	29.1	0.340
ALP (UI/L)	24	38.3	44	74.5	0.021
AST (UI/L)	24	35.63	44	36.05	0.807
CK (UI/L)	24	159.4	42	189.1	0.506

* Comparison between birds’ sex by Student’s *t*-test (two-sample *t*-test). *n*: number of animals tested.

**Table 3:** Comparison of serum biochemical parameters in greater rhea (*R. americana*) and ostriches (*Struthio camelus*).

*Rhea americana*					*Struthio camelus*			
Parameters	Mean	SD	Range	Reference Interval	Mean	SD	Range	Reference Interval
Total protein (g/dL)	4.9	0.57	3.99–6.46	3.4 ± 5.6 ^5^	4.74	0.5	3.5–6	3.9–5.6 ^1^
Albumin (g/dL)	2.56	0.32	1.7–3.3	1.0 ± 2.5 ^5^	1.64	0.485	1.3	0.9–2.2 ^6^
Cholesterol (mmol/L)	6.2	3.7	0.6–16.8	1.94 ±0.1 ^2^	1.67	0.36	1	1.15–2.15 ^6^
Calcium (mmol/L)	3.3	0.52	2.25–5.85	3.85 ± 0.21 ^2^	3.2	0.7	2.3–5.4	2.4–4.8 ^1^
Phosphorus (mmol/L)	1.3	0.21	0.74–1.71	1.9 ± 0.08 ^2^	1.81	0.43	1.19	1.22–2.42 ^6^
Uric acid (µmol/L)	419.2	91.9	225.3–667.5	317. 67 ± 20.82 ^2^	484	92	303–575	351–649 ^1^
Urea (mmol/L)	0.98	0.58	0.02–3.9	0.6 ± 0.30 ^1^	0.64	0.30	0.40–2.8	0.5–0.8 ^1^
				0.8 ^4^				
Creatinine (µmol/L)	29.6	5.64	15.9–45.97	12.0 ^4^	21.3	5.4	12.0–32.5	18.5–24.0 ^1^
ALP (UI/L)	91.5	276.4	10.8–2248.0	171.5 ± 45.9 ^3^	126	60	56–381	69–217 ^1^
AST (UI/L)	38.2	24	12.2–193.3	43.7 ± 7.3 ^2^	321	56	143–471	243–418 ^1^
CK (UI/L)	207.4	224.3	29.2–1464.5	22.3 ± 3.1 ^2^	2667	1041	1268–5954	1648–4894 ^1^
				933.0 ± 269.0 ^3^				

SD: Standard deviation. ^1^ Verstappen et al. [17], ^2^ Reissig et al. [18], ^3^ Palomeque et al. [13], ^4^ Menon et al. [19], ^5^ Ritchie et al. [20], ^6^ Omidi & Ansari nik [21].

## Data Availability

Datasets used and/or analyzed during the current study are available from the corresponding author upon request.
